# Predicting factors for the efficacy of cross-linking for keratoconus

**DOI:** 10.1371/journal.pone.0263528

**Published:** 2022-02-03

**Authors:** Denise Wajnsztajn, Or Shmueli, Ken Zur, Joseph Frucht-Pery, Abraham Solomon

**Affiliations:** Department of Ophthalmology, Hadassah-Hebrew University Medical Center, Jerusalem, Israel; Edith Wolfson Medical Center, ISRAEL

## Abstract

**Purpose:**

To evaluate predictors for success in corneal crosslinking (CXL) for keratoconus in a large cohort and extended follow-up.

**Design:**

A retrospective study based on a prospectively built database.

**Methods:**

Participants underwent CXL for keratoconus from 2007 to 2018. Statistical analysis was performed for patients with at least 1-year follow-up. We analyzed effects of CXL type (Epithelium-on or Epithelium-off and Accelerated (9mW/cm2@10min) or Standard (3mW/cm2@30min)) and pre-operative factors including age, gender, baseline LogMAR visual acuity (LogMAR_pre_), maximal corneal power (Kmax_pre_), pachymetry, refractive and topographic cylinders, spherical equivalent (SE_pre_), mean corneal power (MeanK) and follow-up time on outcome measures. The outcome measures were the final change of Kmax (Delta Kmax) and the final change in LogMAR visual acuity (Delta LogMAR). A more negative Delta Kmax or Delta LogMAR represents a favorable effect of crosslinking.

**Results:**

517 eyes had Kmax results, and 385 eyes had LogMAR results with more than one year follow-up. These eyes were included in the study. The mean follow-up time was 2.29 years. Mean Kmax decreased from 54.07±5.99 diopters to 52.84±5.66 diopters (p<0.001), and Mean LogMAR decreased from 0.28±0.20 to 0.25±0.21 (p<0.001). Non-accelerated epithelium-off CXL resulted in greater flattening of Kmax when compared with other protocols. Visual acuity improvement was similar when comparing different CXL protocols. Multivariate analysis showed four factors associated with negative Delta Kmax: high Kmax_pre_, high SE_pre_, high MeanK_pre,_ and non-accelerated procedure. Multivariate analysis showed three factors associated with negative Delta LogMAR: high LogMAR_pre_, high SE_pre_, and Low MeanK_pre_. After excluding corneas with Kmax_pre_ >65 D or Pachymetry<400 microns, multivariate analysis showed that high Kmax_pre_, high SE_pre_, and non-accelerated CXL were associated with negative Delta Kmax while high LogMAR_pre_ and high SE_pre_ were associated with negative Delta LogMAR.

**Conclusion:**

CXL for keratoconus is a highly effective treatment, as evident by its effects on the outcome measures: Delta Kmax and Delta LogMAR. CXL was more successful in eyes with high Kmax_pre_, high SE_pre_, and high LogMAR_pre, which_ express disease severity. The non-accelerated epithelium-off protocol was associated with greater flattening of corneal curvature but did not show a better effect on visual acuity as compared to the other CXL protocols.

## Introduction

Keratoconus (KC) is a bilateral, progressive corneal ectasia characterized by progressive central or paracentral thinning, protrusion, and irregular astigmatism with a potential for severe visual loss. Onset occurs at puberty and progresses through the 2^nd^ and 3^rd^ decades [1].

Corneal collagen crosslinking (CXL) is a procedure used to strengthen the corneal tissue by creating new covalent bonds within and between amino acid residues in the collagen fibers of the cornea. This increases the biomechanical strength of the keratoconic cornea and halts the progression of ectasia. Corneal collagen crosslinking treatment induces photochemically triggered crosslinks within the collagen network by using a combination of vitamin B_2_ (riboflavin) and 370 nm-wavelength ultraviolet A radiation [2].

Different studies used different outcome measures to evaluate the success of CXL, varying from topographic indices and LogMAR visual acuity to biomechanical and wavefront analysis [3–9]. However, the maximal corneal refractive power (Kmax) is the most utilized parameter to evaluate the efficacy, while the LogMAR is the acceptable parameter to evaluate safety [10]. Most studies report significant success rates following CXL. A meta-analysis of 49 trials, including four randomized controlled trials, reported a mean decrease of 1 diopter in Kmax two years following CXL and improvement in best-corrected distance visual acuity one year following CXL [7]. In another meta-analysis of 75 trials, CXL was demonstrated to be effective with a mean decrease of 0.19 in LogMAR of uncorrected distance visual acuity two years following treatment [4].

The main shortcomings of the current literature on CXL are the small number of trials with more than 100 eyes and short follow-up times, usually of less than two years. Additionally, most studies report outcomes comparing them to the baseline pre-operative measures, and there is a lack of studies evaluating the factors predicting CXL success systematically using a multivariate analysis approach [11].

Knowledge of pre-operative predictors for a successful outcome in CXL may have clinical implications for ophthalmologists when considering the indications for managing KC patients. The purpose of our study is to evaluate pre-operative predictors for success in CXL in a large cohort of KC patients with an extended follow-up period.

## Methods

### Study design and patient selection

This is a retrospective study based on a prospectively built database. The study collected data of keratoconus patients treated by corneal crosslinking (CXL) during the years 2007–2017 at the cornea service of the Department of Ophthalmology at the Hadassah-Hebrew University Medical Center (Jerusalem, Israel).

The study adhered to the declaration of Helsinki. Institutional Review Board (IRB)/Ethics Committee approval was obtained (approval number 18–0221).

Patient’s informed consent was obtained for the treatment, but was waived by the IRB for the participation in this study due to the retrospective and anonymous nature of the data analysis.

All patients who were 18 years old or older at the time of treatment and underwent CXL for progressing KC were included in the study. All gave written consent allowing treatment.

KC was diagnosed based on topographic features such as: an increased area of corneal power surrounded by concentric areas of decreasing power, inferior-superior power asymmetry, skewing of the steepest radial axes above and below the horizonal meridian [1], and/or numeric values such as: Kmax greater than 47.2 D and/or an I-S value greater than 1.4 D [12].

Progression of KC was defined as an increase of at least 1.00 D in topographic Kmax and/or in the refractive cylinder within one year and/or patient’s report of deteriorating visual acuity without any other underlying cause in cases where previous refraction assessment was not possible.

CXL was not performed in patients with active ocular surface disease, history of herpes, stable keratoconus, or pregnant women.

Before CXL, patients were submitted to a complete eye examination with anterior and posterior segment evaluation, intraocular pressure measurement, Schirmer test, and pachymetry (Corneo-Gage PlusTM, Sonogage, Cleveland, OH, USA). Any abnormalities were treated prior to CXL.

Patients were followed up for at least one year after CXL treatment.

Exclusion criteria included insufficient follow-up time (less than one year after CXL) and insufficient data (e.g., Lack of Kmax_pre_ measurement).

### Corneal cross-linking (CXL) procedure

The procedure was done as either epithelium-off or epithelium-on, and in either of these, an accelerated or non-accelerated crosslinking protocol was used.

In epithelium-off, after the application of topical anesthetics, a corneal abrasion of 8 mm diameter was made using a blunt spatula, and isotonic (0.1% riboflavin, 20% dextran (MedioCROSS D, Avedro)) or hypotonic (0.1% riboflavin (MedioCROSS H, Avedro) in corneas <400 microns) riboflavin was applied every 3 minutes for 30 minutes. Pachymetry was measured to ensure a corneal thickness of more than 400 μm prior to Ultra-violet A radiation exposure. In epithelium-on, after instillation of pilocarpine 2%, Ricrolin riboflavin TE (0.1% riboflavin, 15% dextran (Sooft, Italy)) and Oxybuprocaine 0.4% enhanced with BAC were instilled in an interval of 2 minutes for 60 minutes total.

In both cases (epithelium-off or epithelium-on), the cornea was subsequently exposed to a 3- mW/cm^2^ 365-nm UV light source for 30 minutes in the non-accelerated protocol or to a 9- mW/cm^2^ 365-nm UV light source for 10 minutes in the accelerated protocol [2] (UV-X ™ Specifications, IROC, Zurich, Switzerland). In both protocols, a total 5.4 J/cm2 energy was delivered.

Afterward, a 17 mm soft bandage contact lens (Sophlex, Israel) was placed on the cornea in epithelium-off cases for seven days. Patients received topical 0.1% dexamethasone and 0.3% gentamycin four times a day and three times a day, respectively, for a week. After 7 days, when complete epithelialization was evident, the bandage contact lens was removed, and topical fluorometholone was applied three times a day for an additional three months.

Follow-up examinations were performed at 1, 3, 6, and 12 months of the first year and then yearly, including topography, uncorrected and best-corrected visual acuity, and manifest refraction.

### Data collection

Data were collected retrospectively for all patients by the Cornea Service. The demographic and clinical variables documented included: age, gender, follow up period (from treatment, in months), central corneal thickness (pachymetry, in microns, using Corneo-Gage Plus^TM^, Sonogage, Cleveland, OH, USA), accelerated or non-accelerated treatment protocol, epithelium-on or epithelium-off treatment protocol, maximal corneal power before treatment (Kmax_pre_), best-corrected distance visual acuity in LogMAR before treatment (LogMAR_pre_), corneal cylinder before treatment (Cyl_pre_, in diopters), spherical equivalent before treatment (SE_pre_, in diopters), corneal cylinder measured by topography before treatment (TopoCyl_pre_, in diopters), mean corneal power before treatment (meanK_pre_, in diopters).

The data were collected during the period of 2007 through 2018. Visual acuity was tested with a Snellen chart and then converted to a LogMAR visual acuity score. Cyl and SE were recorded after manifest refraction, corneal thickness, Kmax, TopoCyl, and meanK were measured by corneal topography and tomography (EyeSys 2000; EyeSys Vision Inc., Houston, Texas, USA and Pentacam, Oculus Inc, Germany).

The maximal corneal power (referred to as Kmax in our study) was obtained in EyeSys through the axial numeric map provided by the topographer. The highest value was recorded and used for comparison. The Pentacam Kmax was recorded as it appears in the device display.

Patients were followed with the same device used for pre-operative measures. Patients who had EyeSys topography pre-CXL were followed-up with EyeSys, while patients who had a Pentacam scan pre-CXL were followed with Pentacam tomography post-CXL.

Age, gender, and follow-up times were documented in the patient’s electronic medical records.

### Data analysis

The two study outcome measures were changes in Kmax-values (Delta Kmax) and changes in visual acuity (Delta LogMAR) after CXL.

We defined Delta Kmax as the difference between maximal corneal power (in diopters) at the last follow-up of at least one year following treatment (Kmax_last_) and maximal corneal refractive power before CXL (Kmax_pre_).

This difference is defined in the following equation:

Kmax_last_-Kmax_pre_ = Delta Kmax.

A value of 0 or a negative value of Delta Kmax indicates no increase or a decrease in the corneal power, respectively. Thus, a negative value of Delta Kmax indicated a favorable effect of crosslinking.

We defined Delta LogMAR as the difference in visual acuity expressed in LogMAR between the last follow-up of at least one year following treatment (LogMAR_last_) and LogMAR before treatment (LogMAR_pre_).

This difference is defined in the following equation:

LogMAR_last_-LogMAR_pre_ = Delta LogMAR.

A value of 0 or negative values in Delta LogMAR indicates no decrease or an improvement in visual acuity, respectively, thereby demonstrating a favorable effect of crosslinking.

Delta Kmax and Delta LogMAR were analyzed with paired Student’s t-test to evaluate the significance of the change.

We performed ANOVA test to compare the differences in Delta Kmax and Delta LogMAR when using different CXL protocols (Non-accelerated, epithelium-off VS Accelerated, epithelium-off VS Accelerated, Epithelium-on).

We first tested the effects of the independent variables on the outcome measures (Delta Kmax and Delta LogMAR) with univariate analysis. Paired t-test and Pearson correlation (with r coefficient) were used for continuous variables, which included age, LogMAR_pre_, Kmax_pre_, pachymetry before treatment, Cyl_pre_, TopoCyl_pre_, SE_pre_, MeanK_pre_ and follow-up time. Independent samples student’s t-test was used for categorical variables, which included epithelium-on or epithelium-off CXL, Accelerated or Non-Accelerated CXL, and gender. Subsequently, variables that demonstrated a significant effect on either of the outcome measures in the univariate analysis were included in the multivariate analysis using stepwise linear regression analysis.

In order to test the effects of the independent variables on the outcome measures of a more homogenous patient population, we subsequently excluded extremely steep (>65 diopters) or extremely thin (<400 microns) corneas and performed again the univariate and multivariate analyses, respectively, as described above.

Data were analyzed using SPSS version 24.0 (IBM).

## Results

613 eyes of 456 patients underwent CXL at baseline. 517 eyes had Kmax results, and 385 eyes had LogMAR results with more than one year follow up. These eyes were included in the study. The mean follow-up time was 2.29 years (range 1–8 years). Baseline characteristics are presented in Table 1.

**Table 1:** Baseline patient’s characteristics.

Baseline continuous variables	Number of eyes	Median	Mean±SD^1^ (Min-Max)
**Age (years)**	613	26.00	26.46±6.46 (18–65)
**Follow up (months)**	517	20.07	26.40±21.92 (12–115.97)
**Pachymetry (microns)**	581	448.00	444.42±46.33 (307–578)
**Kmax**_**pre**_^**2**^ **(D)**	613	53.00	53.94±5.96 (41.20–83.50)
**LogMAR**_**pre**_ ^**3**^	608	0.22	0.28±0.20 (0–1.30)
**Cyl**_**pre**_^**4**^ **(D)**	578	-4.00	-4.48±2.59 (-14.00–0)
**TopoCyl**_**pre**_^**5**^ **(D)**	613	2.80	2.98±1.76 (0–16.1)
**SE**_**pre**_^**6**^ **(D)**	578	-1.50	-2.42±3.12 (-19.25–4.88)
**MeanK**_**pre**_^**7**^ **(D)**	613	48.10	49.21±5.22 (38–70.65)
**Baseline categorical variables**			
**Gender**	male	393 eyes (290 patients)	
	female	220 eyes (166 patients)	
**Epithelium on/off**	Epithelium-off	514 eyes (84.3%)	
	Epithelium-on	96 eyes (15.7%)	
**Accelerated/non-accelerated**	non-accelerated	175 eyes (28.7%)	
	Accelerated	435 eyes (71.3%)	

Continuous variables are presented by mean, median, standard deviation, and minimum/maximum values.

^1^SD = standard deviation

^2^Kmax_pre_ = maximal corneal power before cross-linking

^3^LogMAR_pre_ = Logarithm of minimal angle of resolution before cross-linking

^4^Cyl_pre_ = refractive cylinder before cross-linking

^5^TopoCyl_pre_ = corneal cylinder before cross-linking as measured by topography

^6^SE_pre_ = Spherical equivalent before cross-linking

^7^MeanK_pre_ = Mean of the twoaxes of corneal astigmatism (K1 and K2) before cross-linking.

### Primary outcome measure 1: Delta Kmax

517 eyes had available Kmax results for a least one year and were included in this analysis. The results show a significant difference between Kmax_last_ of 52.84±5.66 D and Kmax_pre_ of 54.07±5.99 D (p<0.001) with a Delta Kmax of -1.23±3.11 diopter.

Overall, 73.5% of patients had Delta Kmax < -1D (improvement), 4.9% of patients had Delta Kmax > +1D (worsening) and 21.6% of patients had Delta Kmax between -1D and +1D (stable).

The Non-accelerated epithelium-off protocol yielded the most negative Delta Kmax (-2.58±3.81), as compared to the Accelerated, Epithelium-off or Accelerated, Epithelium-on CXL protocols (Table 2; p<0.001). There were not enough patients with non-accelerated epithelium-on protocol to include in this analysis.

**Table 2:** Comparison of Delta Kmax and Delta LogMAR with different crosslinking protocols analyzed with ANOVA (p<0.001).

	Non-accelerated, Epithelium-off	Accelerated, Epithelium-off	Accelerated, Epithelium-on	P-value
N^2^ (%)	175 (28.7%)	339 (55.6%)	96 (15.7%)	NA
Delta Kmax^3^ (Means±SD^5^)	-2.58±3.81	-0.84±2.56	0.25±2.02	<0.001
Delta LogMAR^4^ (Means±SD^5^)	-0.03±0.20	-0.03±0.16	-0.02±0.09	0.876

^1^Kmax = Maximal corneal power

^2^N = number of eyes

^3^Delta Kmax = (maximal corneal power after cross-linking)–(maximal corneal power before cross-linking)

^4^Delta LogMAR = (LogMAR of best-corrected visual acuity after cross-linking)–(LogMAR of best-corrected visual acuity before cross-linking)

^5^SD = standard deviation.

### Delta Kmax: Univariate analysis

**Fig 1:**
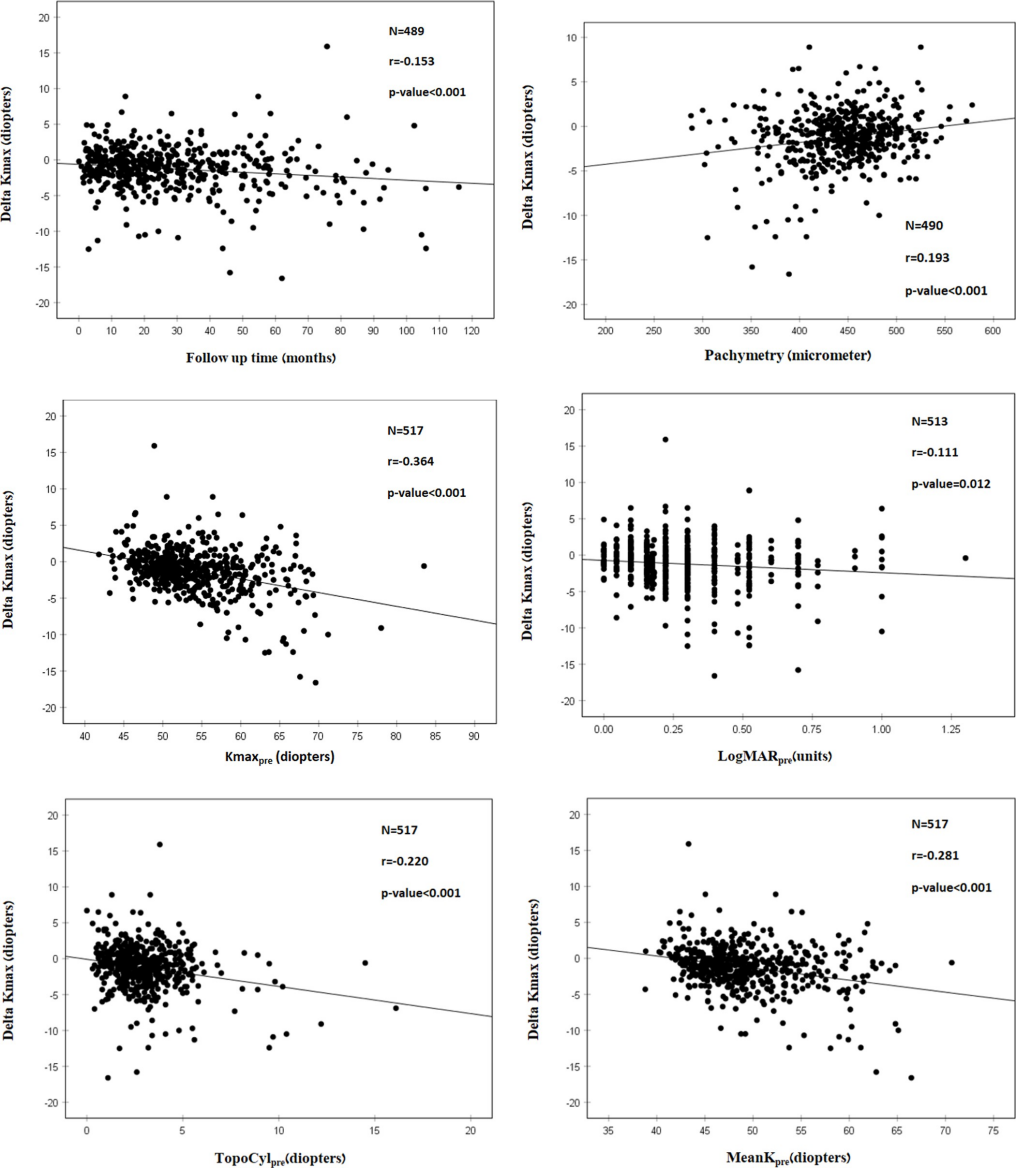
A. Correlation between follow-up time and Delta Kmax^1^. More negative Delta Kmax values were correlated with longer follow-up times, indicating the treatment effect was greater with longer follow-up times. However, the correlation coefficient is low, with r^2^ = -0.153. Thus, although the correlation is significant, it is relatively weak. ^1^Delta Kmax = (maximal corneal power after cross-linking)–(maximal corneal power before cross-linking). ^2^r = Pearson correlation coefficient. B. Correlation between corneal thickness (pachymetry) and Delta Kmax^1^. More negative Delta Kmax values were significantly correlated with thinner corneas. This indicates that a thinner cornea can predict a better treatment outcome. However, the correlation coefficient is low, with r^2^ = 0.193. Thus, although the correlation is significant, it is relatively weak. ^1^Delta Kmax = (maximal corneal power after cross-linking)–(maximal corneal power before cross-linking). ^2^r = Pearson correlation coefficient. C. Correlation between Kmax_pre_^1^ and Delta Kmax^2^. More negative Delta Kmax values were significantly correlated with higher Kmax_pre_ values (more severe baseline keratoconus). However, the correlation coefficient is low with r^3^ = -0.364. Thus, although the correlation is significant, it is relatively weak. ^1^Kmax_pre_ = maximal corneal power before crosslinking. ^2^Delta Kmax = (maximal corneal power after cross-linking)–(maximal corneal power before cross-linking). ^3^r = Pearson correlation coefficient. D. Correlation between LogMAR_pre_^1^ and Delta Kmax^2^. More negative Delta Kmax values were significantly correlated with higher LogMAR_pre_. This indicates that a poorer baseline visual acuity can predict a better treatment outcome. However, the correlation coefficient is low with r^3^ = -0.111. Thus, although the correlation is significant, it is relatively weak. ^1^LogMAR_pre_ = Logarithm of minimal angle of resolution before crosslinking. ^2^Delta Kmax = (maximal corneal power after cross-linking)–(maximal corneal power before cross-linking). ^3^r = Pearson correlation coefficient. E. Correlation between TopoCyl_pre_^1^ and Delta Kmax^2^. More negative Delta Kmax values were significantly correlated with higher TopoCyl_pre_. This indicates that higher baseline corneal astigmatism can predict better treatment outcomes. However, the correlation coefficient is low with r^3^ = -0.220. Thus, although the correlation is significant, it is relatively weak. ^1^TopoCyl_pre_ = corneal cylinder before crosslinking as measured by topography. ^2^Delta Kmax = (maximal corneal power after cross-linking)–(maximal corneal power before cross-linking). ^3^r = Pearson correlation coefficient. F. Correlation between MeanK_pre_^1^and Delta Kmax^2^. Higher MeanK_pre_ is correlated with a more negative Delta Kmax and can predict better treatment outcomes. However, the correlation coefficient is low, with r^3^ = -0.281. Thus, although the correlation is significant, it is relatively weak. ^1^MeanK_pre_ = Mean of the two axes of corneal astigmatism (K1 and K2) before crosslinking. ^2^Delta Kmax = (maximal corneal power after cross-linking)–(maximal corneal power before cross-linking). ^3^r = Pearson correlation coefficient.

Correlation analysis demonstrated a significant correlation between a longer follow-up time (r = -0.153; P<0.001) and negative Delta Kmax values, indicating the treatment effect was greater the further it was measured from the time of treatment (Fig 1A).

A significant correlation was also found between a more negative Delta Kmax and thinner corneas (r = 0.193; P<0.001) (Fig 1B), higher Kmax_pre_ (r = -0.364; P<0.001) (Fig 1C), higher LogMAR_pre_ (r = -0.111; P = 0.012) (Fig 1D), higher TopoCyl_pre_ (r = -0.220; P<0.001) (Fig 1E), higher MeanK_pre_ (r = -0.281; P<0.001) (Fig 1F) and higher SE_pre_ (r = -0.091; P = 0.045) (correlations are presented in Fig 1A–1F and Table 3).

**Table 3:** Univariate analysis of variables affecting Delta Kmax.

Variants	N^2^	Pearson Correlation (r^3^)	P-value
**Age**	517	0.02	0.642
**Sex:**	326	-	0.087*
**Men**			
**Women**	191		
**Follow-up**	489	-0.153	P<0.001
**Pachymetry**	490	0.193	P<0.001
**Non-Accelerated**	165	-	
**Accelerated**	352		P<0.001*
**Epithelium Off**	440	-	
**Epithelium On**	75		P<0.001*
**Kmax**_**pre**_ ^**4**^	517	-0.364	P<0.001
**LogMAR**_**pre**_ ^**5**^	513	-0.111	0.012
**Cyl**_**pre**_ ^**6**^	488	0.049	0.277
**SE**_**pre**_ ^**7**^	488	-0.091	0.045
**TopoCyl**_**pre**_ ^**8**^	517	-0.220	P<0.001
**MeanK**_**pre**_ ^**9**^	517	-0.281	P<0.001

The correlation between continuous variables and Delta Kmax was analyzed with regression analysis.

*Comparison of the categorical variables was tested with independent samples t-test.

^1^Delta Kmax = (maximal corneal power after cross-linking)–(maximal corneal power before cross-linking)

^2^N = number of patients

^3^r = Pearson correlation coefficient

^4^Kmax_pre_ = maximal corneal power before cross-linking

^5^LogMAR_pre_ = Logarithm of minimal angle of resolution before cross-linking

^6^Cyl_pre_ = refractive cylinder before cross-linking

^7^SE_pre_ = Spherical equivalent before cross-linking

^8^TopoCyl_pre_ = corneal cylinder before cross-linking as measured by topography

^9^MeanK_pre_ = Mean of the two axes of corneal astigmatism (K1 and K2) before cross-linking.

**Fig 2:**
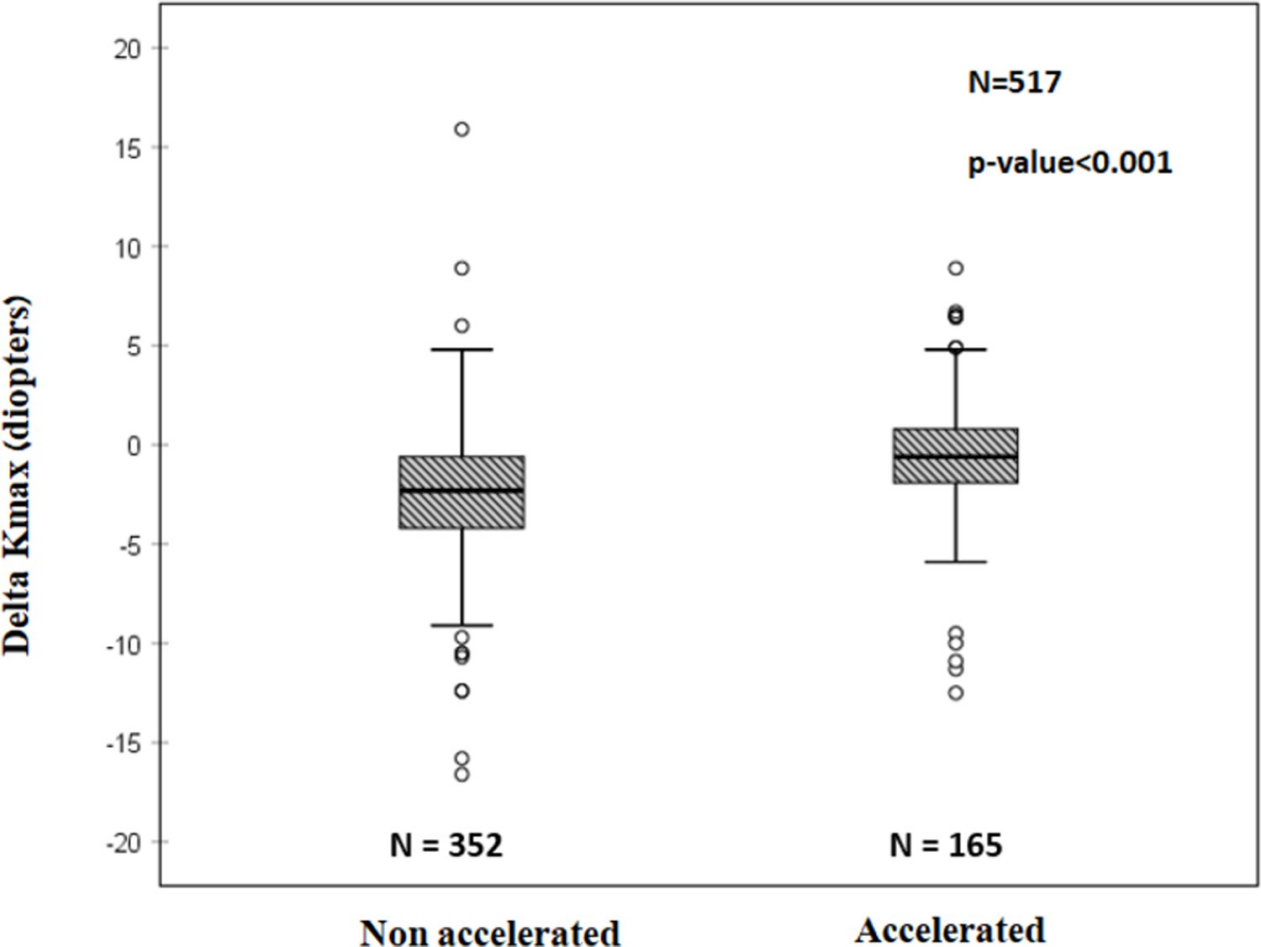
Delta Kmax^1^ with non-accelerated and accelerated CXL^2^ protocols. Paired t-test showed significantly more mean negative Delta Kmax values with the non-accelerated compared to the accelerated protocol (P<0.001), indicating a greater efficacy for the non-accelerated protocol. ^1^Delta Kmax = (maximal corneal power after cross-linking)–(maximal corneal power before cross-linking). ^2^CXL = Cross-linking.

More negative Delta Kmax values were observed with non-accelerated CXL protocol (P<0.001) compared to the accelerated CXL protocol (Fig 2, Table 3).

**Fig 3:**
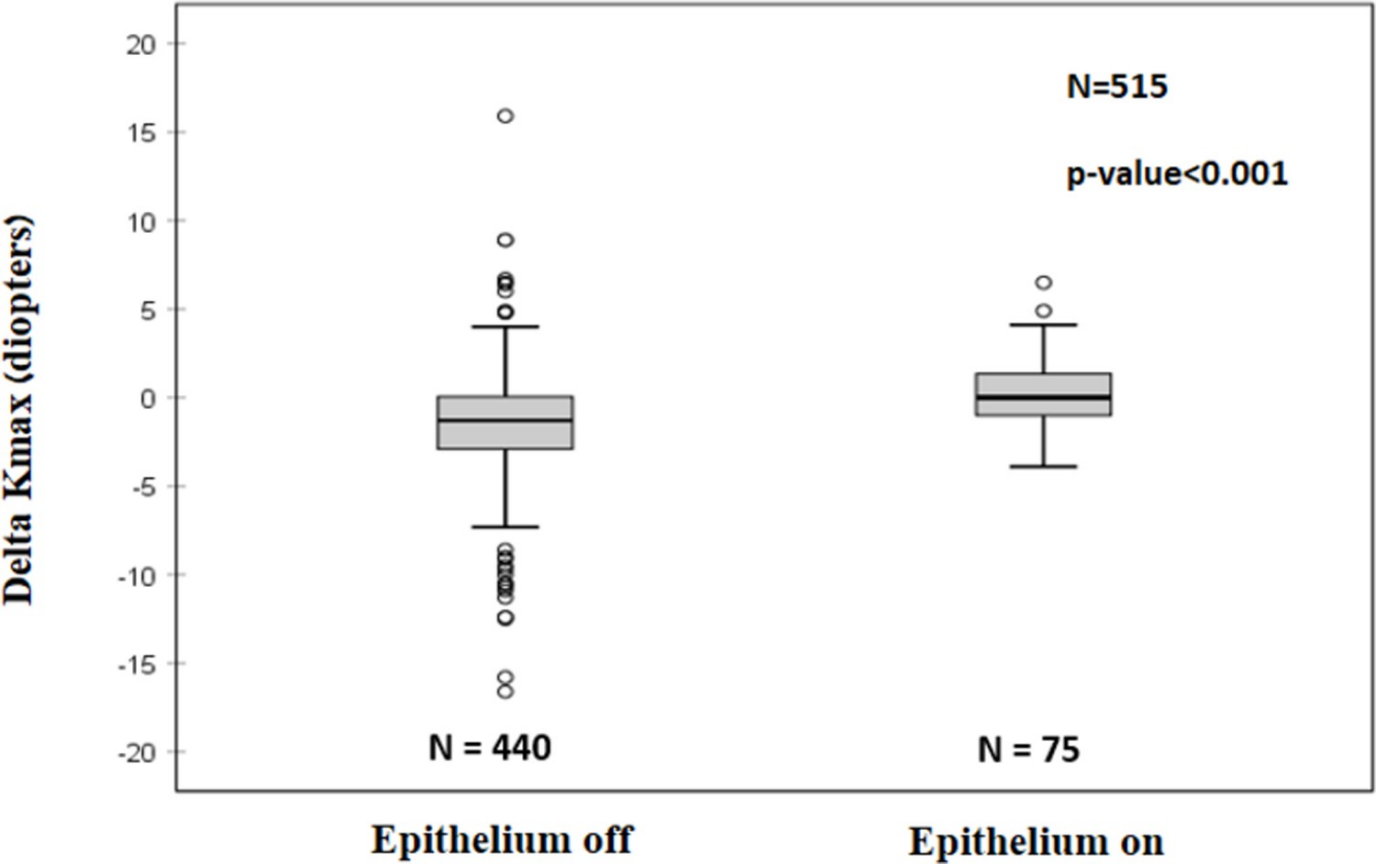
Delta Kmax^1^ with epithelium-off and epithelium-on CXL^2^ protocols. Paired t-test showed significantly more mean negative Delta Kmax value with the epithelium-off protocol (P<0.001), indicating a greater efficacy for the epithelium-off compared with the epithelium-on protocol. ^1^Delta Kmax = (maximal corneal power after cross-linking)–(maximal corneal power before cross-linking). ^2^CXL = Cross-linking.

More negative Delta Kmax values were observed with epithelium-off CXL protocol (P<0.001), compared to the epithelium-on CXL protocol (Fig 3, Table 3).

### Delta Kmax: A multivariate analysis

Four variables remained significant in multivariate analysis (Model’s R^2^ = 20.5%; Table 4). By order of significance level, these include Kmax_pre_ (P<0.001), accelerated/non-accelerated CXL protocol (P<0.001), SE_pre_ (P = 0.014), and MeanK_pre_ (P = 0.015).

**Table 4:** Multivariate analysis of variables affecting Delta Kmax.

variants	N^2^	P-value	Β
**Follow-up**	441	0.961	-0.002
**Pachymetry**		0.181	-0.070
**Non-Accelerated**			0.228
**Accelerated**		**p<0.001**	
**Epithelium Off**			0.057
**Epithelium On**		0.218	
**Kmax**_**pre**_ ^**3**^		**p<0.001**	-0.619
**LogMAR**_**pre**_ ^**4**^		0.185	0.072
**SE**_**pre**_ ^**5**^		**0.014**	-0.111
**TopoCyl**_**pre**_ ^**6**^		0.183	-0.066
**MeanK**_**pre**_ ^**7**^		**0.015**	0.315

Variables that were significant in the univariate analysis were included in multivariate analysis using stepwise approach linear regression.

^1^Delta Kmax = (maximal corneal power after crosslinking)–(maximal corneal power before crosslinking)

^2^N = number of patients

^3^Kmax_pre_ = maximal corneal power before crosslinking

^4^LogMAR_pre_ = Logarithm of minimal angle of resolution before crosslinking

^5^SE_pre_ = Spherical equivalent before crosslinking

^6^TopoCyl_pre_ = corneal cylinder before crosslinking as measured by topography

^7^MeanK_pre_ = Mean of the two axes of corneal astigmatism (K1 and K2) before crosslinking.

After exclusion of extremely steep (Kmax >65 D) or thin corneas (pachymetry < 400 microns) (363 eyes left available for analysis), three variables remained significantly correlated with Delta Kmax in multivariate analysis. By order of significance level, these include accelerated/non-accelerated CXL protocol (P<0.001), SE_pre_ (P<0.001), and Kmax_pre_ (P<0.001). (Model’s R^2^ = 21%; S1 and S2 Tables).

### Primary outcome 2: Delta LogMAR

385 eyes had available LogMAR results for a least one year and were included in this analysis. The results show a significant difference between LogMAR_pre_ of 0.28±0.20 and LogMAR_last_ of 0.25±0.21 (p<0.001) with a Delta LogMAR of -0.031±0.17.

There was no significant difference in Delta LogMAR when comparing different CXL protocols (Non-accelerated, epithelium-off VS Accelerated, epithelium-off VS Accelerated, Epithelium-on; Table 2; p<0.001). There were not enough patients with non-accelerated epithelium-on protocol to include in this analysis.

### Delta LogMAR: Univariate analysis

**Fig 4:**
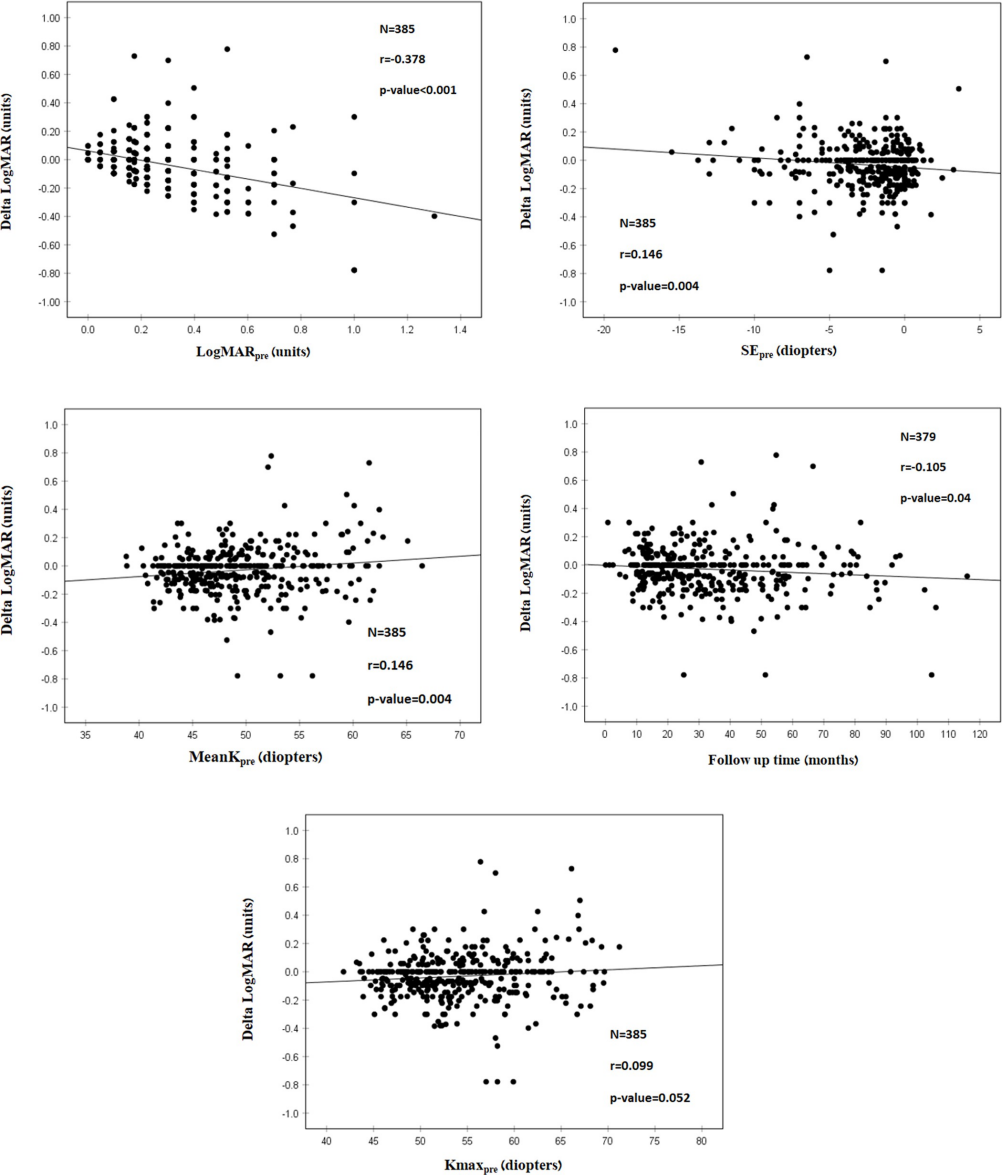
A. Correlation between LogMAR_pre_^1^ and Delta LogMAR^2^. More negative Delta LogMAR was correlated with higher LogMAR_pre_. r^3^ = -0.378. These results indicate that poorer baseline visual acuity can predict better treatment outcomes with a greater improvement of visual acuity. ^1^LogMAR_pre_ = Logarithm of minimal angle of resolution before crosslinking. ^2^Delta LogMAR = (Logarithm of minimal angle of resolution after crosslinking)–(Logarithm of minimal angle of resolution before crosslinking). ^3^r = Pearson correlation coefficient. B. Correlation between MeanK_pre_^1^ and Delta LogMAR^2^. More negative Delta LogMAR was correlated with LOWER MeanK_pre_, indicating LOWERbaseline MeanK correlates with lesser visual acuity improvement. However, the correlation coefficient is low, with r^3^ = 0.146. Thus, although the correlation is significant, it is relatively weak. ^1^LogMAR_pre_ = Logarithm of minimal angle of resolution before crosslinking. ^2^Delta LogMAR = (Logarithm of minimal angle of resolution after crosslinking)–(Logarithm of minimal angle of resolution before crosslinking). ^1^MeanK_pre_ = Mean of the two axes of corneal astigmatism (K1 and K2) before crosslinking. ^2^Delta LogMAR = (Logarithm of minimal angle of resolution after crosslinking)–(Logarithm of minimal angle of resolution before crosslinking). ^3^r = Pearson correlation coefficient. C. Correlation between SE_pre_^1^and Delta LogMAR^2^. More negative Delta LogMAR was correlated with HIGHER SE_pre_, indicating that HIGHER baseline SE correlates with greater visual acuity improvement and more effective treatment. However, the correlation coefficient is low with r^3^ = -0.126. Thus, although the correlation is significant, it is weak. ^1^SE_pre_ = Spherical equivalent before crosslinking. ^2^Delta LogMAR = (Logarithm of minimal angle of resolution after crosslinking)–(Logarithm of minimal angle of resolution before crosslinking). ^3^r = Pearson correlation coefficient. D. Correlation between Follow up time and Delta LogMAR^1^. More negative Delta LogMAR was correlated with a longer follow-up time. This indicates longer follow-up time correlates with greater visual acuity improvement and more effective treatment. However, the correlation coefficient is low, with r^2^ = -0.105. Thus, although the correlation is significant, it is weak. ^1^Delta LogMAR = (Logarithm of minimal angle of resolution after crosslinking)–(Logarithm of minimal angle of resolution before crosslinking). ^2^r = Pearson correlation coefficient. E. Correlation between Kmaxpre^1^ and Delta LogMAR^2^. More negative Delta LogMAR was correlated with HIGHER Kmax_pre_. This indicates HIGHER baseline Kmax measurement correlates with greater visual acuity improvement and more effective treatment. The correlation coefficient is low, with r^3^ = 0.099. With borderline significance (p = 0.052). ^1^Kmax_pre_ = maximal corneal power before crosslinking. ^2^Delta LogMAR = (Logarithm of minimal angle of resolution after crosslinking)–(Logarithm of minimal angle of resolution before crosslinking). ^3^r = Pearson correlation coefficient.

In this analysis, 4 variables were found to be significantly correlated to Delta LogMAR (Table 5 and Fig 4): LogMAR_pre_ (r = -0.378; P<0.001) (Fig 4A), SE_pre_ (r = -0.126; P = 0.016) (Fig 4B), MeanK_pre_ (r = 0.146; P = 0.004) (Fig 4C) and follow up time (r = -0.105; P = 0.040) (Fig 4D). In addition, Kmax_pre_ had borderline significance (r = 0.099; P-value = 0.052) (Fig 4E).

**Table 5:** Effects of independent variables on Delta LogMAR–Univariate analysis.

variants	N^2^ (eyes)	Pearson Correlation (r^3^)	P-value
**age**	**385**	0.034	0.512
**Sex:**		-	0.880*
**men**	**244**		
**women**	**141**		
**Follow-up**	**379**	-0.105	0.040
**pachymetry**	**367**	-0.061	0.246
**Non-Accelerated**	**139**	-	
**Accelerated**	**246**		0.508*
**Epithelium Off**	**324**	-	
**Epithelium On**	**59**		0.544*
**Kmax**_**pre**_ ^**4**^	**385**	0.099	0.052
**LogMAR**_**pre**_ ^**5**^	**385**	-0.378	P<0.001
**Cyl**_**pre**_ ^**6**^	**367**	-0.028	0.596
**SE**_**pre**_ ^**7**^	**367**	-0.126	0.016
**TopoCyl**_**pre**_ ^**8**^	**385**	-0.034	0.5
**MeanK**_**pre**_ ^**9**^	**385**	0.146	0.004

The correlation between continuous variables and Delta LogMAR was tested with regression analysis.

*Comparison of the categorical variables was tested with independent samples t-test.

^1^Delta LogMAR = (LogMAR after cross-linking)–(LogMAR before cross-linking)

^2^N = number of eyes

^3^r = Pearson correlation coefficient

^4^Kmax_pre_ = maximal corneal power before cross-linking

^5^LogMAR_pre_ = Logarithm of minimal angle of resolution before cross-linking

^6^Cyl_pre_ = refractive cylinder before cross-linking

^7^SE_pre_ = Spherical equivalent before cross-linking

^8^TopoCyl_pre_ = corneal cylinder before cross-linking as measured by topography

^9^MeanK_pre_ = Mean of the two axes of corneal astigmatism (K1 and K2) before cross-linking.

### Delta LogMAR: A multivariate analysis

Three variables remained significant in the multivariate analysis) Model’s R^2^ = 29.4%; Table 6(. These include LogMAR_pre_ (P<0.001), SE_pre,_ (P<0.001) and MeanK_pre_ (P = 0.001).

**Table 6:** Multivariate analysis of variables affecting Delta LogMAR.

Variants	N^2^	P-value	β
**Follow-up**	367	0.062	-0.088
**Kmax**_**pre**_ ^**3**^		0.896	-0.073
**LogMAR**_**pre**_ ^**4**^		P<0.001	-0.626
**SE**_**pre**_ ^**5**^		P<0.001	-0.189
**MeanK**_**pre**_ ^**6**^		0.001	0.417

Variables that were significant in the univariate analysis were included in multivariate analysis using stepwise approach linear regression.

^1^Delta LogMAR = (LogMAR after crosslinking)–(LogMAR before crosslinking)

^2^N = number of patients

^3^Kmax_pre_ = maximal corneal power before crosslinking

^4^LogMAR_pre_ = Logarithm of minimal angle of resolution before crosslinking

^5^SE_pre_ = Spherical equivalent before crosslinking

^6^MeanK_pre_ = Mean of the two axes of corneal astigmatism (K1 and K2) before crosslinking.

After exclusion of extremely steep (Kmax >65 D) or thin corneas (pachymetry < 400 microns) (290 eyes left available for analysis), two variables were significantly correlated with Delta LogMAR in multivariate analysis. These include LogMAR_pre_ (P<0.001) and SE_pre,_ (P<0.001) (Model’s R^2^ = 26.3%; S3 and S4 Tables).

## Discussion

The present study analyzed the effects of pre-operative predictors for successful results for corneal crosslinking for keratoconus in a large database of patients with a long-term follow-up period. We used two main outcome measures: the change in Kmax and the change in the visual acuity over the follow-up period.

For eyes with at least 1-year follow-up following CXL, our study demonstrated significant improvements of Delta Kmax (-1.23 D) and Delta LogMAR (-0.031).

When comparing the different CXL protocols using ANOVA, Non-accelerated epithelium-off CXL resulted in greater flattening of Kmax when compared with the other protocols.

In our multivariate analysis, corneal flattening effect post-CXL, was significantly affected by higher Kmax_pre_, higher SE_pre_, higher MeanK_pre,_ and non-accelerated CXL procedure.

We also demonstrated that an improvement of visual function post-CXL was significantly affected by a higher LogMAR_pre_, a higher SE_pre,_ and a lower MeanK_pre_.

After exclusion of extremely steep (Kmax >65 D) or thin corneas (pachymetry < 400 microns), higher Kmax_pre_, higher SE_pre_ and non-accelerated CXL procedure remained significantly correlated with Delta Kmax, and LogMAR_pre_ and SE_pre_ remained significantly correlated with Delta LogMAR in multivariate analysis (S2 and S4 Tables).

Overall, these results show that in patients with more advanced disease, CXL might have a more potent effect.

Like our study, previous studies have demonstrated a similar decrease in Kmax and LogMAR following CXL [4–9].

In our study, gender and adult age (all of the patients were above 18 years) were not found to be predictors of Delta Kmax or Delta LogMAR. These findings are in concordance with current literature [13, 14]. One study investigated 96 eyes one year following CXL and compared two age groups: above and below 30 years [15]. The older group showed significantly greater improvement in Kmax but not in LogMAR. Koller and colleagues, however, found that age above 35 years and visual acuity better than 20/25 were predictors of worsening of visual acuity, while a high Kmax and female gender were predictors for treatment failure (increase in Kmax_last_) [10]. We believe our results better represent the real effect of age on the outcome since it was analyzed as a continuous variable (and not dichotomously as in those studies).

In univariate analysis, longer follow-up correlated with a more negative Delta Kmax but had no significance in multivariate analysis. CXL aims to successfully stabilize the cornea, avoiding the disease progression, and the reduction in Kmax is a secondary effect. O’Brart et al. [9] showed that the 36 eyes treated with CXL showed a progressive decrease in Kmax along seven years of follow-up. This was followed by a significant hyperopic shift of +3.00 D or greater in over 10%. In addition, there are reports of significant progressive corneal flattening over time with no underlying cause [16] and associated with corneal scar [17]. This long-term flattening is not well understood, and it is particularly worrisome after entering the hyperopic range, being considered a CXL complication [16]. Interestingly, follow-up time was not significantly correlated with Delta LogMAR in our study. This is beneficial as keratoconus patients, who are highly dependent on visual aids, will not suffer from refractive fluctuations along the time.

In univariate analysis, thinner corneas (lower pachymetry) correlated with a more negative Delta Kmax. This is in agreement with a study by Yam et al. [18]. However, in the multivariate analysis, the corneal thickness did not come out as a significant predictor. Likewise, no significant correlation was demonstrated for the corneal thickness on the Delta LogMAR. Toprak et al. found a correlation between thinner corneas and a decrease in Kmax following CXL [15]. In that study, the patients were divided into two groups: above and below 450 microns of corneal thickness. When comparing both groups, the thinner corneas demonstrated more significant improvement in Kmax, with no change in LogMAR. Our study analyzed corneal thickness as a continuous variable rather than categorical, and it had a larger sample size when compared to previous works. We believe this better reflects the overall clinical effects in our cohort of patients. In addition, corneal thickness correlates with keratoconus severity. Thus, eyes with thinner corneas are the eyes with more severe topographic characteristics (such as Kmax_pre_ and TopoCyl_pre_) at baseline. Stepwise linear regression grades the variables by the strength of their correlation to the outcome. It is probable that other parameters were better correlated with the outcome, and hence, pachymetry was found non-significant on multivariate analysis.

Epithelium-off CXL was correlated with significantly more negative Delta Kmax compared to epithelium-on CXL outcome in the univariate analysis. This is in agreement with previous studies [2, 19, 20]. However, in multivariate analysis, this effect was not evident. This difference reflects a controversy in the literature regarding the difference between these techniques. The corneal epithelial cells are connected by tight junctions, which play a vital role in epithelial functions [21]. It is hypothesized that these tight junctions limit the diffusion of riboflavin into the stroma and lessen its effects during the epithelium-on CXL procedure [22]. Nevertheless, our study did not find a difference between these techniques in multivariate analysis. Epithelium-off or epithelium-on techniques had no significant effect on Delta LogMAR in our study, both in univariate and multivariate analysis. These results are in agreement with a recent meta-analysis including eight studies, demonstrating no differences in Kmax and corrected distance visual acuity between eyes treated with epithelium-off and epithelium-on CXL after one year of follow-up [23].

In univariate and multivariate analysis, non-accelerated CXL was correlated with significantly more negative Delta Kmax compared to accelerated CXL. Furthermore, when we compared the different CXL protocols using ANOVA, Non-accelerated epithelium-off CXL resulted in greater flattening of Kmax when compared with the other protocols.

In a recent meta-analysis including 22 studies and 1158 eyes, the non-accelerated CXL provided a better corneal flattening [24]. This was also confirmed by another meta-analysis, including 11 trials [25], further supporting our results. In addition, the combination of non-accelerated with epithelium off CXL has proven to be the most efficacious protocol in our cohort regarding corneal Delta Kmax. However, the accelerated protocol has other advantages, such as shorter treatment time and better patient comfort, making it a more appealing method for many clinicians.

In univariate and multivariate analysis, higher Kmax_pre_ was correlated with significantly more negative Delta Kmax. In 2009, Koller et al. [10] showed that eyes with Kmax equal or greater than 58D had higher chances of failed treatment after one year, and inclusion criteria below this number would have decreased the failure rate (Kmax_last_ increase above 1.0D) from 7.6% to 2.8%.

However, in 2011, Koller T et al. [13] found a significant flattening effect in Kmax after one year of follow-up in corneas with a Kmax_pre_ of 54 D or higher. In their study, based on their previous report, the authors concluded that their results create a relatively narrow band of maximum success between 54.00 D and 58.00 D of Kmax, in which a flattening rate greater than 50% was accompanied by a failure rate of less than 1%. Other studies support our findings. Greenstein et al. [14] showed that eyes with a Kmax_pre_ equal or higher than 55.0 D were 5.4 times (95% CI, 2.1–14.0) more likely to have topographic flattening equal or higher than 2.0 D. Sloot et al. l [26] also observed that in corneas with higher Kmax (≥ 58 D), the flattening effect was more significant than in less advanced keratoconus corneas.

Lower Kmax_pre_ had a borderline correlation with more negative Delta LogMAR in univariate analysis. Sloot and colleagues [26] also identified a significant improvement in corrected distance visual acuity after CXL in the group with lower Kmax_pre_ (<58D), while this difference was not significant in the higher Kmax_pre_ group. However, while demonstrating a significant correlation with Delta LogMAR on univariate analysis, Kmax_pre_ failed to correlate significantly to delta LogMAR on multivariate analysis in our study. Although we found a weak correlation between Kmax_pre_ and Delta LogMAR, this might not be clinically relevant. The visual acuity improvement after CXL is believed to be related to the decrease in high order aberrations secondary to the change of the physical properties of the cornea and not necessarily to corneal shape [4]. We believe our study better reflects the clinical picture as we tested Kmax_pre_ as a continuous and not as a categorical variable, as was performed in previous studies, making the analysis more accurate.

Worse baseline visual acuity, represented in our study as higher LogMAR_pre_, was significantly correlated with better visual acuity following treatment in univariate and multivariate analysis. Toprak [15] showed that KC eyes with vision ≤ 20/40 pre-CXL had significantly greater visual improvement following CXL. Greenstein [14] observed that eyes with ≤20/40 were 5.9 times (95% confidence interval [CI], 2.2–6.4) more likely to improve 2 Snellen lines or more. Moreover, Godefrooij [27] found that worse pre-treatment corrected distance visual acuity was found to be the sole independent factor predicting an improvement in corrected distance visual acuity one year after CXL. Wisse et al. [11], in a multivariate analysis, showed that specifically, a low visual acuity predicts visual improvement at a year follow-up. All of the above studies support our findings. Interestingly, Koller and colleagues identified that a pre-operative corrected distance visual acuity better than 20/25 was a significant risk factor for losing two or more Snellen lines with an OR of 18.18 [10].

Higher baseline spherical equivalent (SE) correlated with greater improvement of Kmax in univariate analysis and visual acuity in multivariate analysis. However, it should be noted that SE is not a good indicator of KC severity and may be misleading since most of the abnormality in KC is contributed by high cylinder values, and the spherical refractive error is smaller in magnitude. In addition, the spherical refractive error is highly influenced by the axial length. The effect of the SE on the outcome measures was included in our analysis, similar to previous studies. Our study contrasted with the results of Wisse et al. [11] that found no correlation between SE and changes in both corneal power and visual acuity post-CXL after uniand multivariate analysis. We found it relevant to analyze if SE would be a predictor of CXL success because the final SE can be influenced by the CXL effect. While in some studies, SE appears to be stable [5, 28] it can be affected in the long term, as was reported by O’Brart and colleagues [9], which found a significant change of SE 7 years post-CXL (mean gain of +0.78D).

Our study has three significant advantages over the current literature. First, the sample size is significantly larger than most previous studies, with 613 eyes included. Second, we followed patients for an extended period of up to 8 years follow-up. Third, this is one of the few studies that present a systematic analysis of multiple predictors for CXL success using univariate and multivariate statistical tools.

Our study also has limitations. First, although it is based on a prospectively built database, it is a retrospective analysis, and therefore there was some missing data for a small proportion of our cohort. Second, the study cohort lacked control eyes not treated with CXL. However, our study was not designed to evaluate the efficacy of this procedure for KC. Since the efficacy of CXL has been demonstrated in many studies for years before our study, it would be unethical not to perform this procedure in KC patients who needed it [5, 6, 9, 29].

In addition, our study includes cases from 2007, when CXL was less popular and well known and was not yet introduced to the Israeli National Health basket until 2014. This means that cases performed during the early years might have been of a more advanced KC, whereas cases performed in later years might have been more moderate. As the procedure gained acceptance, popularity, and with the growing awareness of the efficacy of CXL, more community ophthalmologists and optometrists referred more KC patients and at earlier stages. Lastly, it comprises mostly Middle-Eastern patients who are known to have more aggressive progression than other populations [30].

In conclusion, we have shown in our study that advanced keratoconus corneas might benefit better from the late effects of CXL, such as a decrease in Kmax and improvement of visual acuity than early KC corneas. Still, early detection and treatment are critical for the patient’s benefit, and treatment should not be delayed to achieve a higher effect. Our multivariate analysis demonstrated the baseline factors that can predict CXL success. These are higher Kmax_pre_, higher SE_pre_, lower MeanK_pre,_ and non-accelerated protocol, which correlate with improvement in Kmax. Higher LogMAR_pre_ (strongest correlation), higher SE_pre,_ and lower MeanK_pre_ predict better improvement in LogMAR visual acuity. These findings can serve a vital clinical need to predict and advise on crosslinking outcomes to both patients and clinicians.

## Supporting information

S1 Table Univariate analysis of variables affecting Delta Kmax^1^ after exclusion of extremely steep (>65 D) or thin (<400 microns) corneas. The correlation between continuous variables and Delta Kmax was analyzed with regression analysis. *Comparison of the categorical variables was tested with independent samples t-test. ^1^Delta Kmax = (maximal corneal power after cross-linking)–(maximal corneal power before cross-linking); ^2^N = number of patients;^3^r = Pearson correlation coefficient;^4^Kmax_pre_ = maximal corneal power before cross-linking; ^5^LogMAR_pre_ = Logarithm of minimal angle of resolution before cross-linking; ^6^Cyl_pre_ = refractive cylinder before cross-linking; ^7^SE_pre_ = Spherical equivalent before cross-linking; ^8^TopoCyl_pre_ = corneal cylinder before cross-linking as measured by topography; ^9^MeanK_pre_ = Mean of the two axes of corneal astigmatism (K1 and K2) before cross-linking.
(DOCX)

S2 Table Multivariate analysis of variables affecting Delta Kmax (N^2^ = 363) after exclusion of extremely steep (>65 D) or thin (<400 microns) corneas. Variables that were significant in the univariate analysis were included in multivariate analysis using stepwise approach linear regression. ^1^Delta Kmax = (maximal corneal power after cross-linking)–(maximal corneal power before cross-linking); ^2^N = number of eyes; ^3^Kmax_pre_ = maximal corneal power before cross-linking; ^4^LogMAR_pre_ = Logarithm of minimal angle of resolution before cross-linking; ^5^SE_pre_ = Spherical equivalent before cross-linking; ^6^TopoCyl_pre_ = corneal cylinder before cross-linking as measured by topography; ^7^MeanK_pre_ = Mean of the two axes of corneal astigmatism (K1 and K2) before cross-linking.
(DOCX)

S3 Table Effects of independent variables on Delta LogMAR after exclusion of extremely steep (>65 D) or thin (<400 microns) corneas–Univariate analysis. The correlation between continuous variables and Delta LogMAR was tested with regression analysis. *Comparison of the categorical variables was tested with independent samples t-test. ^1^Delta LogMAR = (LogMAR after cross-linking)–(LogMAR before cross-linking); ^2^N = number of eyes; ^3^r = Pearson correlation coefficient; ^4^Kmax_pre_ = maximal corneal power before cross-linking; ^5^LogMAR_pre_ = Logarithm of minimal angle of resolution before cross-linking; ^6^Cyl_pre_ = refractive cylinder before cross-linking; ^7^SE_pre_ = Spherical equivalent before cross-linking; ^8^TopoCyl_pre_ = corneal cylinder before cross-linking as measured by topography; ^9^MeanK_pre_ = Mean of the two axes of corneal astigmatism (K1 and K2) before cross-linking.
(DOCX)

S4 Table Multivariate analysis of variables affecting Delta LogMAR (N^2^ = 283) after exclusion of extremely steep (>65 D) or thin (<400 microns) corneas. Variables that were significant in the univariate analysis were included in multivariate analysis using stepwise approach linear regression. ^1^Delta LogMAR = (LogMAR after cross-linking)–(LogMAR before cross-linking); ^2^N = number of eyes; ^3^Kmax_pre_ = maximal corneal power before cross-linking; ^4^LogMAR_pre_ = Logarithm of minimal angle of resolution before cross-linking; ^5^SE_pre_ = Spherical equivalent before cross-linking; ^6^MeanK_pre_ = Mean of the two axes of corneal astigmatism (K1 and K2) before cross-linking.
(DOCX)

S1 Data (XLSX)

## Abbreviations and acronyms used

Kmax_pre_: maximal corneal curvature before crosslinking
Kmax_last_: maximal corneal power after crosslinking at last follow up
LogMAR_pre_: Logarithm of minimal angle of resolution before crosslinking
LogMAR_last_: Logarithm of minimal angle of resolution after crosslinking at last follow up
Cyl_pre_: refractive cylinder before crosslinking
TopoCyl_pre_: corneal cylinder before crosslinking as measured by topography
MeanK_pre_: Mean of the two axes of corneal astigmatism (K1 and K2) before crosslinking
SE_pre_: Spherical equivalent before crosslinking
CXL: Cross-linking
KC: Keratoconus
SD: Standard deviation
