# Supplementary material for: Predicting factors for the efficacy of cross-linking for keratoconus
Source: PLoS One. 2022 Feb 3;17(2):e0263528. doi: 10.1371/journal.pone.0263528 (PMC8812864; doi:10.1371/journal.pone.0263528)
Supplement: S1 Table — The correlation between continuous variables and Delta Kmax was analyzed with regression analysis. *Comparison of the categorical variables was tested with independent samples t-test. 1Delta Kmax = (maximal corneal power after cross-linking)–(maximal corneal power before cross-linking); 2N = number of patients;3r = Pearson correlation coefficient;4Kmaxpre = maximal corneal power before cross-linking; 5LogMARpre = Logarithm of minimal angle of resolution before cross-linking; 6Cylpre = refractive cylinder before cross-linking; 7SEpre = Spherical equivalent before cross-linking; 8TopoCylpre = corneal cylinder before cross-linking as measured by topography; 9MeanKpre = Mean of the two axes of corneal astigmatism (K1 and K2) before cross-linking. (DOCX) [file pone.0263528.s001.docx]

**Supplementary material**

**Table 1s.** **Univariate analysis of variables affecting Delta Kmax^1^ after exclusion of extremely steep (>65 D) or thin (<400 microns) corneas**

| **P-value** | **Pearson Correlation (r^3^)** | **N^2^** | **Variants** |
| --- | --- | --- | --- |
| 0.20 | 0.06 | 398 | **Age** |
| 0.71 | - | 241 | **Sex: Men** |
|  |  | 157 | **Women** |
| P<0.001 | -0.180 | 378 | **Follow-up** |
| 0.03 | 0.105 | 398 | **Pachymetry** |
|  | - | 126 | **Non-Accelerated** |
| P<0.001^*^ |  | 272 | **Accelerated** |
|  | - | 335 | **Epithelium Off** |
| P<0.001^*^ |  | 62 | **Epithelium On** |
| P<0.001 | -0.280 | 398 | **Kmax_pre_^4^** |
| 0.116 | -0.079 | 395 | **LogMAR_pre_^5^** |
| 0.48 | 0.036 | 383 | **Cyl_pre_^6^** |
| 0.003 | -0.150 | 383 | **SE_pre_^7^** |
| P<0.001 | -0.179 | 398 | **TopoCyl_pre_^8^** |
| P<0.001 | -0.179 | 398 | **MeanK_pre_^9^** |

**Table 1s.** **Univariate analysis of variables affecting Delta Kmax^1^ after exclusion of extremely steep (>65 D) or thin (<400 microns) corneas.** The correlation between continuous variables and Delta Kmax was analyzed with regression analysis. *Comparison of the categorical variables was tested with independent samples t-test.

^1^Delta Kmax=(maximal corneal power after cross-linking) – (maximal corneal power before cross-linking); ^2^N=number of patients;^3^r= Pearson correlation coefficient;^4^Kmax_pre_=maximal corneal power before cross-linking; ^5^LogMAR_pre_= Logarithm of minimal angle of resolution before cross-linking; ^6^Cyl_pre_=refractive cylinder before cross-linking; ^7^SE_pre_=Spherical equivalent before cross-linking; ^8^TopoCyl_pre_= corneal cylinder before cross-linking as measured by topography; ^9^MeanK_pre_=Mean of the two axes of corneal astigmatism (K1 and K2) before cross-linking.
