# Supplementary material for: Predicting factors for the efficacy of cross-linking for keratoconus
Source: PLoS One. 2022 Feb 3;17(2):e0263528. doi: 10.1371/journal.pone.0263528 (PMC8812864; doi:10.1371/journal.pone.0263528)
Supplement: S2 Table — Variables that were significant in the univariate analysis were included in multivariate analysis using stepwise approach linear regression. 1Delta Kmax = (maximal corneal power after cross-linking)–(maximal corneal power before cross-linking); 2N = number of eyes; 3Kmaxpre = maximal corneal power before cross-linking; 4LogMARpre = Logarithm of minimal angle of resolution before cross-linking; 5SEpre = Spherical equivalent before cross-linking; 6TopoCylpre = corneal cylinder before cross-linking as measured by topography; 7MeanKpre = Mean of the two axes of corneal astigmatism (K1 and K2) before cross-linking. (DOCX) [file pone.0263528.s002.docx]

**Supplementary material**

**Table 2s.** **Multivariate analysis of variables affecting Delta Kmax^1^ after exclusion of extremely steep (>65 D) or thin (<400 microns) corneas**

| **β** | **P-value** | **Variants** |
| --- | --- | --- |
| 0.007 | 0.898 | **Follow-up** |
| -0.04 | 0.941 | **Pachymetry** |
| 0.304 |  | **Non-Accelerated** |
|  | **p<0.001** | **Accelerated** |
| 0.063 |  | **Epithelium Off** |
|  | 0.217 | **Epithelium On** |
| -0.135 | **p<0.001** | **Kmax_pre_^3^** |
| -0.184 | **p<0.001** | **SE_pre_^5^** |
| -0.089 | 0.074 | **TopoCyl_pre_^6^** |
| 0.075 | 0.512 | **MeanK_pre_^7^** |

**Table 2s.** **Multivariate analysis of variables affecting Delta Kmax (N^2^=363) after exclusion of extremely steep (>65 D) or thin (<400 microns) corneas.** Variables that were significant in the univariate analysis were included in multivariate analysis using stepwise approach linear regression.

^1^Delta Kmax=(maximal corneal power after cross-linking) – (maximal corneal power before cross-linking); ^2^N=number of eyes; ^3^Kmax_pre_=maximal corneal power before cross-linking; ^4^LogMAR_pre_= Logarithm of minimal angle of resolution before cross-linking; ^5^SE_pre_=Spherical equivalent before cross-linking; ^6^TopoCyl_pre_= corneal cylinder before cross-linking as measured by topography; ^7^MeanK_pre_=Mean of the two axes of corneal astigmatism (K1 and K2) before cross-linking.
