# Supplementary material for: Predicting factors for the efficacy of cross-linking for keratoconus
Source: PLoS One. 2022 Feb 3;17(2):e0263528. doi: 10.1371/journal.pone.0263528 (PMC8812864; doi:10.1371/journal.pone.0263528)
Supplement: S3 Table — The correlation between continuous variables and Delta LogMAR was tested with regression analysis. *Comparison of the categorical variables was tested with independent samples t-test. 1Delta LogMAR = (LogMAR after cross-linking)–(LogMAR before cross-linking); 2N = number of eyes; 3r = Pearson correlation coefficient; 4Kmaxpre = maximal corneal power before cross-linking; 5LogMARpre = Logarithm of minimal angle of resolution before cross-linking; 6Cylpre = refractive cylinder before cross-linking; 7SEpre = Spherical equivalent before cross-linking; 8TopoCylpre = corneal cylinder before cross-linking as measured by topography; 9MeanKpre = Mean of the two axes of corneal astigmatism (K1 and K2) before cross-linking. (DOCX) [file pone.0263528.s003.docx]

**Supplementary material**

**Table 3s. Effects of independent variables on Delta LogMAR^1^ after exclusion of extremely steep (>65 D) or thin (<400 microns) corneas- Univariate analysis**

| **P-value** | **Pearson Correlation (r^3^)** | **N^2^ (eyes)** | **variants** |
| --- | --- | --- | --- |
| 0.055 | 0.111 | **299** | **age** |
| 0.896^*^ | - | **181** | **Sex: men** |
|  |  | **118** | **women** |
| 0.015 | -0.142 | **293** | **Follow-up** |
| 0.883 | 0.009 | **299** | **pachymetry** |
|  | - | **107** | **Non-Accelerated** |
| 0.409^*^ |  | **192** | **Accelerated** |
|  | - | **248** | **Epithelium Off** |
| 0.544^*^ |  | **50** | **Epithelium On** |
| 0.951 | 0.004 | **299** | **Kmax_pre_^4^** |
| P<0.001 | -0.455 | **299** | **LogMAR_pre_^5^** |
| 0.355 | 0.054 | **290** | **Cyl_pre_^6^** |
| 0.002 | -0.183 | **290** | **SE_pre_^7^** |
| 0.589 | -0.031 | **299** | **TopoCyl_pre_^8^** |
| 0.398 | 0.049 | **299** | **MeanK_pre_^9^** |

**Table 3s. Effects of independent variables on Delta LogMAR after exclusion of extremely steep (>65 D) or thin (<400 microns) corneas – Univariate analysis.** The correlation between continuous variables and Delta LogMAR was tested with regression analysis.

*Comparison of the categorical variables was tested with independent samples t-test.

^1^Delta LogMAR=(LogMAR after cross-linking) – (LogMAR before cross-linking); ^2^N=number of eyes; ^3^r=Pearson correlation coefficient; ^4^Kmax_pre_=maximal corneal power before cross-linking; ^5^LogMAR_pre_= Logarithm of minimal angle of resolution before cross-linking; ^6^Cyl_pre_= refractive cylinder before cross-linking; ^7^SE_pre_=Spherical equivalent before cross-linking; ^8^TopoCyl_pre_= corneal cylinder before cross-linking as measured by topography; ^9^MeanK_pre_=Mean of the two axes of corneal astigmatism (K1 and K2) before cross-linking.
