# Supplementary material for: Predicting factors for the efficacy of cross-linking for keratoconus
Source: PLoS One. 2022 Feb 3;17(2):e0263528. doi: 10.1371/journal.pone.0263528 (PMC8812864; doi:10.1371/journal.pone.0263528)
Supplement: S4 Table — Variables that were significant in the univariate analysis were included in multivariate analysis using stepwise approach linear regression. 1Delta LogMAR = (LogMAR after cross-linking)–(LogMAR before cross-linking); 2N = number of eyes; 3Kmaxpre = maximal corneal power before cross-linking; 4LogMARpre = Logarithm of minimal angle of resolution before cross-linking; 5SEpre = Spherical equivalent before cross-linking; 6MeanKpre = Mean of the two axes of corneal astigmatism (K1 and K2) before cross-linking. (DOCX) [file pone.0263528.s004.docx]

**Supplementary material**

**Table 4s.** **Multivariate analysis of variables affecting Delta LogMAR^1^ after exclusion of extremely steep (>65 D) or thin (<400 microns) corneas**

| **Β** | **P-value** | **Variants** |
| --- | --- | --- |
| -0.078 | 0.115 | **Follow-up** |
| -0.437 | P<0.001 | **LogMAR_pre_^4^** |
| -0.014 | P<0.001 | **SE_pre_^5^** |

**Table 4s.** **Multivariate analysis of variables affecting Delta LogMAR (N^2^=283) after exclusion of extremely steep (>65 D) or thin (<400 microns) corneas.** Variables that were significant in the univariate analysis were included in multivariate analysis using stepwise approach linear regression.

^1^Delta LogMAR=(LogMAR after cross-linking) – (LogMAR before cross-linking); ^2^N=number of eyes; ^3^Kmax_pre_=maximal corneal power before cross-linking; ^4^LogMAR_pre_= Logarithm of minimal angle of resolution before cross-linking; ^5^SE_pre_=Spherical equivalent before cross-linking; ^6^MeanK_pre_=Mean of the two axes of corneal astigmatism (K1 and K2) before cross-linking.
